# Prison Reform

**Published:** 1921-08-13

**Authors:** 


### THE PUBLIC WELL-BEING.

### Prison Reform.

In the year 1889 there appeared in The Hospital an article entitled "The Intermediate Sentence." This was, we believe, the first account in any English newspaper of that development of prison reform which was going on in America, and was seen most conspicuously in the Elmira Reformatory. The assumption underlying this reform was that imprisonment might be made not merely punitive to the criminal and deterrent to others, but absolutely remedial and educative to the culprit. Adopted in England under the name of the Borstal system, this assumption has been fully justified. As in Elmira, the system is applied only to young criminals on their first conviction, and its value depends largely on a fairly long sentence being inflicted on the offender. It recognises that a first crime may be the result merely of the high spirits which make many a lad and girl impatient of control, or, at the worst, of some sudden temptation. It realises also that though there may be, as Lombroso holds, a distinctly criminal type of humanity, all criminals do not belong to it. Acting on these principles, our authorities have developed a method of dealing with young criminals, the results of which are most encouraging.

Perhaps the supreme evidence that the criminal is not outside humanity is to be found in the tablet which was recently unveiled at Dartmoor, in memory of ex-convicts who laid down their lives in the war. But further evidence of patriotism on the part of these failures of the State is to be found in the fact that during the war prisoners made over twenty million articles for Government Departments. The question of prison labour has aroused much discussion. Many members of the industrial classes resent the competition of convict labour in the market. But the work done by prisoners is not really competitive with free labour. Rather is it a part of their training for honest work in the outside world, and, apart from the moral point of view, every man restored to honourable work is an asset and an economy to the nation. That the Borstal system has justified itself is proved by the fact that only 27 per cent. of those who have come under it since the Act of 1908 have been reconvicted. A method which saves nearly three-fourths of those who are dealt with is certainly worth having. The value of the work of convicts is steadily rising, and in 1919-1920 it amounted to a little over £15 per head. On their discharge the earnings of convicts are not handed directly to them for fear that old associates of an evil kind may get hold of the discharged man, but are entrusted to the Church Army or some other of the associations that care for ex-prisoners. According to the discretion of those at the head of these organisations, the money may be employed for the maintenance of the man until he obtains employment, or it may be used for the purchase of tools to enable him to resume his former trade. These details have to be settled by that wise humanity which, we hope, is now dominating our prison system.

Even more considerate than the Borstal system is that of Probation, by which the culprit escapes punishment for his first offence unless he falls again. It is a delicate matter to say in what cases this lenity should be shown, and it has not yet become common. There is always the risk of it being abused and giving temporary immunity to the determined criminal. But with young offenders it may save them from ever having a prison record. With the regular criminal, the man who commits a fresh crime as soon as he is released from prison, medical investigation is of the first importance. Undoubtedly many habitual criminals are defectives, for whom the greatest kindness would be a life sentence, but it would be wrong to inflict this without a careful inquiry into the psychology of the offender. The problems of the prison are not yet solved, but how great an improvement there has been attained in our methods of dealing with these anti-social elements of the population may be inferred from the fact that the recent book of Sir Evelyn Ruggles-Brise on *The English Prison System* was printed at the Convict Printing Press at Maidstone.

